# Biomechanical evaluation of individual 3D-printed vertebrae

**DOI:** 10.1186/s42490-026-00107-w

**Published:** 2026-03-06

**Authors:** Florian Metzner, Stefan Schleifenbaum, Christoph-Eckhard Heyde, Nicolas Heinz von der Höh

**Affiliations:** 1 ZESBO - Center for Research on Musculoskeletal Systems, Faculty of Medicine, Leipzig University, Semmelweisstraße 14, 04103 Leipzig, Germany; 2 Department of Orthopedic Surgery, Traumatology and Plastic Surgery, University of Leipzig Medical Center, Leipzig, Germany; 3 Fraunhofer Institute for Machine Tools and Forming Technology, Dresden, Germany

**Keywords:** Cancellous bone, 3D-printing, Lattice, Additive manufacturing, Stereolithography, Compressive test, Bone, Osteoporosis, Bone density

## Abstract

**Background:**

Personalized 3D-printed bone models are becoming increasingly popular in clinical care. Common applications include the visualization of idiopathic deformities or complex joint fractures. Functionalizing such printed replicas in terms of individual mechanical properties holds great potential for clinical training and research but is challenging due to the complexity of the bone structure. This study aims at developing a parametrizable structure as a substitute for spongious bone by simplifying 3D reconstruction and printing.

**Methods:**

43 vertebrae from 6 body donors aged 86.8 ± 7.8 years were examined. Each spine underwent a clinical computed tomography scan. Cylindrical samples (Ø6 × 12 mm) were randomly taken from the left or right side of the vertebral body using a core drill in the superior-inferior direction. Specific software was used for determining the volumetric Hounsfield units of the spongious bone in each vertebral hemisphere. In parallel, a parametric hexagonal grid structure was designed using engineering software. All rods within the lattice have a variable length L and a fixed diameter of t = 0.4 mm. By varying the ratio t/L, six different porosities were defined. For each of these, five cylindrical lattice samples (diameter/length = 1/2) from two different synthetic resins were manufactured using the stereolithography printing process. All samples were mechanically characterized by uniaxial compressive testing. Curve fitting based on power functions (y = ax^b^) allowed the determination of correlations between mechanical parameters and Hounsfield units (bone) as well as the lattice parameter t/L (3D-printed lattice). Finally, three vertebrae with varying bone quality were printed with their respected parameterized lattice and evaluated by comparing the axial screw pullout forces of the human and the respective printed bones.

**Results:**

There is a significant correlation between the mechanical properties of the bone specimens and the determined Hounsfield units. Furthermore, the mechanical properties of the lattice can be excellently described by the ratio t/L. The printed vertebrae showed pull-out forces similar to those of osteoporotic bone.

**Conlusion:**

The mechanical behavior of vertebral human spongious bone can be well reproduced by a 3D-printed generic lattice structure. Patient-specific bone models can be generated by integrating the parameterizable lattice structure into the specific bone contours. These models can help in improving patient care, for instance by enabling highly realistic surgical approaches for particularly complex anatomies.

## Background

Patient-specific bone models are often used as visual support in the planning and execution of complex operations [1–4]. They are used, for example, in oral and maxillofacial surgery [5], spinal surgery [6–8] and trauma surgery [9–11]. Both operating time and blood loss can be significantly reduced by using individual 3D-printed models [6] and they can be produced with minimal shape deviations [12–14]. However, the mechanical properties of the printed models vary greatly depending on shape, printing process, printing settings (part orientation, infill), model material and the interaction between the above parameters [15–17]. Functionalizing patient-specific anatomical models in terms of their mechanical properties holds promising application potential, not only in medical training for learning surgical techniques [4, 18], but also in biomechanical research as a reproducible test material with individual characteristics [4].

The extrusion process, for example, makes it very easy to adjust the internal infill pattern and wall thickness of the models. Some research groups used this method and carried out a qualitative evaluation of the material properties by having experienced orthopedic surgeons carry out various surgical activities (drilling, sawing, screw implantation) and evaluating the haptics using questionnaires [19, 20]. Although the models can be produced in a simple and cost-effective manner, their subjective approach prevents them from providing reliable evidence about the actual mechanical behavior of the model bones or the infill structure. A different approach is the fabrication of real trabecular architecture. For this purpose, the geometry information was reconstructed from micro-computed tomography (µCT) data and produced in different scales utilizing various 3D-printing processes. Both uniaxial compression tests and screw pull-out tests were performed for analyzing the mechanical behavior and the printability [21, 22]. Current model approaches are not practicable for manufacturing patient-specific bone models capable of replicating the mechanical behavior of the original bone, as either their mechanical properties were not objectively determined or they are based on µCT data, which cannot be performed on patients due to the high radiation intensity.

Indivdualized bone models with realistic and valid mechanical behavior which can be replicated multiple times could enhance clinical practise and research, like pre-surgical planning or evaluation of treatment strategies, by minimizing the dependence on human body donors and their great inter- and intraindividual differences. Given the high clinical relevance regarding degenerative bone loss and its implications on spinal fixation this work aims in mechanically characterizing vertebral spongious bones via compressive tests and comparing their mechanical parameters with their local Hounsfield units (HU) from diagnostic computed tomography (CT). This data is then used for generating a lattice structure mimicking the mechanical behavior of the spongious bone, which can be parameterized by adapting the lattice dimensions to the individual mechanical behavior of the corresponding bones. Whole vertebrae will finally be replicated to analyze their interactions with pedicle screws and compare them against human bones.

This leads to two hypotheses. Based on diagnostic CT data, a lattice structure with bone-like mechanical material behavior can be generated and specifically adapted to the required mechanical strength. Individual bones can be reproduced by substituting the spongious bone inside the vertebral body with the hexagonal lattice structure, allowing them to interact realistically with implanted pedicle screws.

## Methods

The methodology is structured into two distinct phases. The first phase involves the collection of comparative data from human donor specimens through uniaxial compression testing and axial pull-out tests of pedicle screws. Subsequently, the second phase encompasses a detailed description of the lattice design, its mechanical characterization, and the comparative evaluation of 3D-printed vertebral bones.

### Human bone characterization

**Fig. 1:**
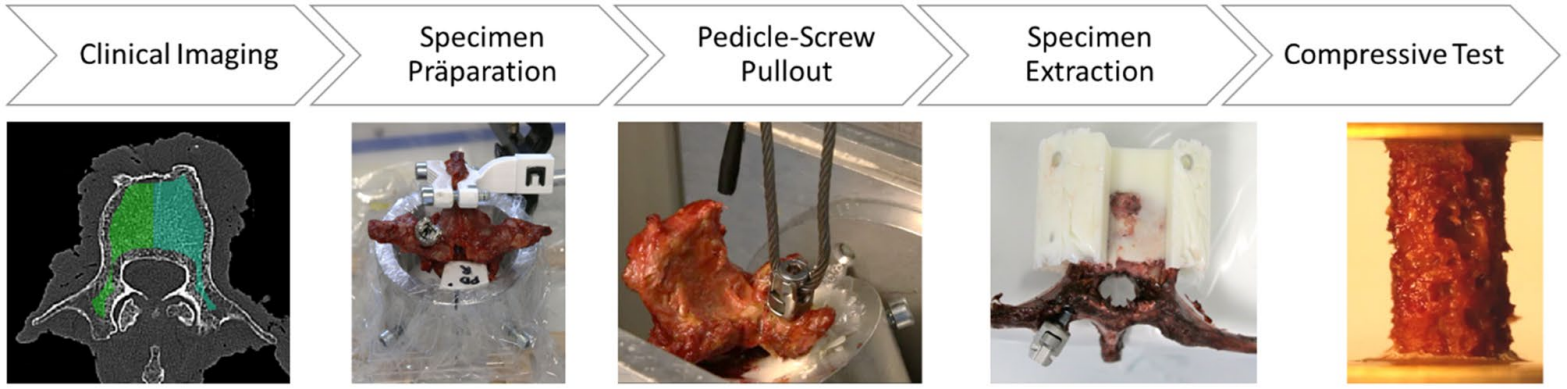
Testing protocol for data acquisition frum human donor tissues

43 vertebrae from 6 human body donors (2 females, 4 males) aged 86.8 ± 7.8 (mean ± standard deviation) years were obtained in fresh and anatomically unfixed condition from levels T7 to L5. All body donors gave their informed and written consent to the donation of their bodies for teaching and research purposes while alive. Being part of the body donor program regulated by the Saxonian Death and Funeral Act of 1994 (third section, paragraph 18 item 8), institutional approval for the use of the post-mortem tissues of human body donors was obtained from the Institute of Anatomy, University of Leipzig (ethical approval No. 129/21-ck). The authors declare that all experiments were conducted according to the principles of the Declaration of Helsinki. All bones were stored fresh frozen at -80 °C until further preparation. CT-scans (Voltage: 120 kV; slice thickness 1 mm; pixel spacing ranging from 0.39 to 0.51 mm; Fig. 1,) as well as Dual-Energy-X-Ray (DXA) scans were conducted on the frozen whole spines. Before testing, all vertebrae were thawed one time to separate each bone from surrounding soft tissues and other bony anatomy. Afterwards the individual bones were freezed again at -80° C until testing.

On the test day, the vertebrae were thawed overnight at 4° C and then a polyaxial pedicle screw (M.U.S.T. Pedicle Screw, Medacta International, Castel San Pietro, Switzerland) was randomly instrumented on one side using the classic trajectory with the freehand technique. This involved pre-drilling with a 2.5 mm diameter and tapping using the respective tools. Each vertebra was aligned in aluminum cylinders using 3D-printed clamps and spacers to align the transverse plane of the vertebra orthogonal to the cylinder axis. The vertebral body was then embedded using a cold-curing cast resin system (RenCast FC 52/53, Huntsman Advanced Materials, Basel, Switzerland). Additional fixing screws were embedded to prevent the casting material from shifting and twisting within the cylinder (Fig. 1, Specimen Preparation). Now the aluminum cylinder got aligned and secured with screws. This is done by inserting a K-wire into the cannulated screw to visualize the longitudinal axis of the screw. A wire rope was then looped into the screw head and connected to the machine’s crosshead using the matching locking screw (Fig. 1, Pedicle-Screw Pullout). The whole fixture was mounted on a xy-table within the testing machine (Allroundline Z10, Zwick/Roell GmbH & Co. KG, Ulm, Germany), equipped with a 2.5 kN lead cell, to eliminate transverse loads. All pull-out tests were carried out with a preload of 5 N and a test speed of 5 mm/min according to ASTM F543 [23] to determine the maximum pull-out force F_max_. Testing was stopped after 60% of F_max_ had been reached.

The embedded vertebra was then extracted from the cylinder and the 3D-printed spacer removed (Fig. 1, Specimen extraction). The spacer forms a free space above the vertebra which minimizes tool wear and also creates a flat surface below the vertebra, enabling the vertebra to be aligned in a cranio-caudal direction within the stationary drilling machine.

**Fig. 2:**
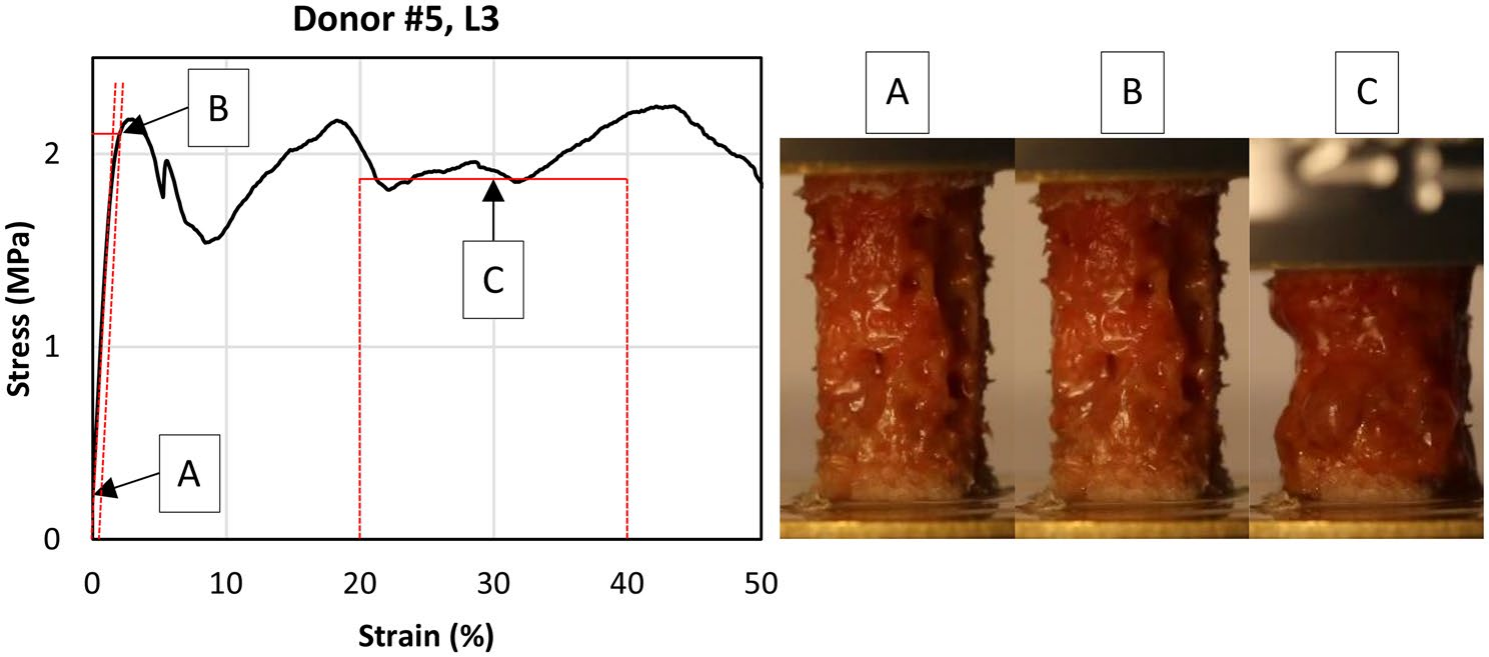
Exemplary stress-strain-curve of a compressive bone specimen from the second lumbar vertebrae of donor #5 (left). The corresponding sample is shown on the right-hand side at that moment when each mechanical parameter is determined. The compressive modulus E is determined at A, the compressive stress σ_0.2_ at B and the plateau stress σ_p_ at C

A drill core of Ø6 mm is extracted via a tenon cutter (FAMAG Series 1616, FAMAG Werkzeugfabrik GmbH & Co. KG, Remscheid, Germany) after explanting the pedicle screw. The cylinder was then cut to a 12 mm length using a band saw (EXAKT 310, EXAKT Advanced Technologies GmbH, Norderstedt, Germany) equipped with a specially designed and 3D-printed cutting device [24]. Brass plates were glued to the end faces of each specimen with cyanoacrylate to minimize end-artifacts (Fig. 1, Compressive Test). Subsequent uniaxial compression testing was also performed using the above-mentioned testing machine, whereby the test load was applied via polished stainless steel test platens. A hysteresis loop between 0.16 MPa and 0.33 MPa is applied as preconditioning. These limits come from pre-tests and are set to avoid irreversible damage to the samples. Afterwards, the samples were tested to a maximal compression of 40%. The determined parameters are Modulus E (maximum slope in the quasilinear range; Fig. 2A), compressive stress σ_y_ (failure stress at 0.2% offset of the E; Fig. 2B) and the plateau stress σ_p_ (mean value of all stress values in the 20–40% strain range; Fig. 2C) [24, 25]. Between all steps, the samples were stored in 0.9% saline solution to prevent dehydration. Each vertebra was tested within one day.

The determined mechanical parameters were then correlated with the corresponding HU from the CT data of their respective vertebral hemisphere. HU is determined using segmentation software (Mimics Innovation Suite V.23, Materialise, Leuven, Belgium) in accordance with the preliminary work of [26]. A filled mask is created for each vertebra using the threshold method. The created mask is then thinned using the Erode function so that a new mask is created containing only cancellous bone tissue. Next, this mask is divided into its left and right sides and the average HU of each mask was exported.

### Modeling and characterizing the lattice structure

**Fig. 3:**
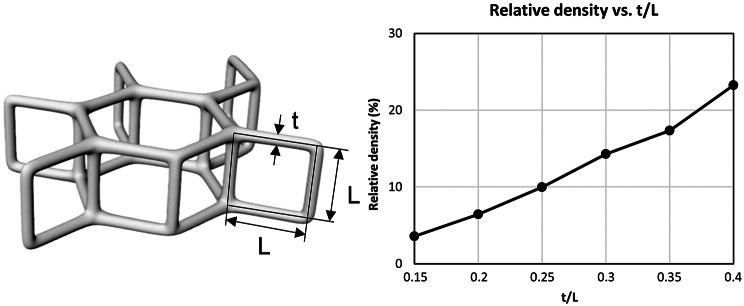
Unit-cell of the hexagonal lattice (left). All struts within the lattice have the same length L and a constant strut diameter t of 0.4 mm. The dependence of the relative density and the ratio t/L is shown on the right side

As described by Gibson [27], hexagonal lattice structures are considered useful for mimicing vertebral trabecular bone, especially for low bone quality. Given the clinical relevance of degenerative bone loss we chose a hexagonal lattice as a simplification for recreating the complex architecture of the cancellous bone within the vertebrea. It was created using the computer-aided-design (CAD) software (Rhino 7, Robert McNeel & Associates, Seattle, WA, USA) and consists of equilateral and equiangular hexagons in one plane, the corners of which are in turn connected with vertical bars between the individual planes. The lattice can thus be clearly defined and customized by a single parameter which is the ratio of strut thickness t and strut length L (Fig. 3).

The average trabecular thickness in lumbar vertebral bodies is approximately 0.12 mm with an average bone volume fraction of 8.15% [28]. Such small struts cannot be reliably printed by stereolithography (SLA). Initial tests determined a minimum printable strut thickness of t = 0.4 mm for the SLA-process. This was done by producing cylindrical test specimens with increasing rod thickness, starting at 0.2 mm, on the SLA printer (Form 3B, Formlabs, Sommerville, MA, USA). The test specimens were then analyzed for printing defects. Since the average trabecular thickness of human cancellous bone in the vertebral body is around 0.1 mm, the smallest rod diameter of 0.4 mm that could be reproducibly printed was selected.

**Fig. 4:**
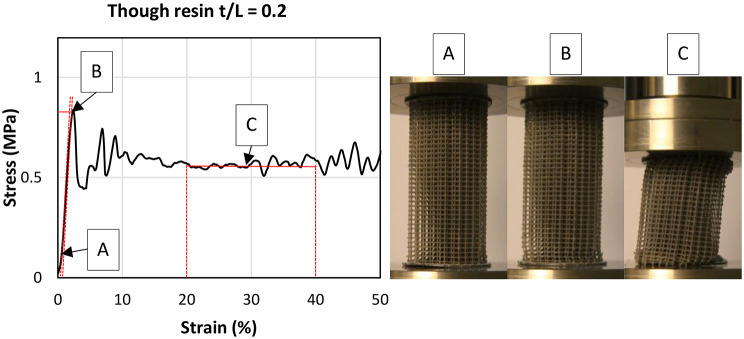
Exemplary stress-strain-curve of a compressive lattice made of Tough2000 V2 resin with t/L = 0.2 (left). The corresponding sample is shown on the right-hand side at that moment when each mechanical parameter is determined. The compressive modulus E is determined at A, the compressive stress σ_0.2_ at B and the plateau stress σ_p_ at C

Cylindrical specimens with a diameter to length ratio of 1:2 were printed for the mechanical characterization (analogous to the bone specimens; Fig. 4 right) [15, 24, 29, 30]. The specimen diameter is generally defined to be ten times larger than the largest lattice spacing. This ensures the structural integrity of the sample [29]. Mechanical stability of the structure is therefore ultimately defined by the ratio of t/L (strut diameter/strut length) and is therefore independent of the selected t. Changing t/L means a variation in the solid content and thus a change in the structural behavior. The relationship between t/L and the resulting relative density (solid content) of the lattice structure is shown in (Fig. 3, right). The grid is fused with 1 mm thick plates at the end faces of each cylinder for preventing end artefacts during the compression tests [31]. Additionally, these plates provide an attachment surface for support structures during printing. Six samples of each of the selected t/L ratios were printed from each of two resins (Clear V4 and Tough2000 V2, Formlabs, Sommerville, MA, USA). The material characteristics of the resins are shown in Table 1.

**Table 1:** Tensile properties of the used resin materials given by the manufacturer (Formlabs, Sommerville, MA, USA)

Material	Elastic modulus (GPa)	Ultimate tensile strength (MPa)	Elongation at break (%)
Clear V4	2.8	65	6
Tough2000 V2	2.2	46	48

One sample from each sample group (*N* = 6) was used for a preliminary test, so that a total of 30 samples from each material were available for characterizing the lattice structure [30]. Characterization of the 3D-printed specimens followed the same procedure as the human bone compressive tests (Fig. 4).

The parameterization of the grid structure is based on the mathematical relationships between HU and the mechanical parameters of the human print samples (1), as well as the relationship between t/L and the mechanical properties (2) of the printed grid samples. All correlations are carried out by curve fitting of the experimental data by means of power law relations. Parameterization can be performed via two equations and one unknown for each of the three mechanical parameters (E, σ_y_, σ_p_). The relationship (3) is obtained by inserting and rearranging Eqs. (1) and (2).

1 $$y=a{\cdot\left(HU\right)}^{b}$$

2 $$y=u{\cdot\left(\raisebox{1ex}{$t$}\!\left/\!\raisebox{-1ex}{$L$}\right.\right)}^{v}$$

3 $$\raisebox{1ex}{$t$}\!\left/\!\raisebox{-1ex}{$L$}\right.=\sqrt[v]{\frac{a\cdot{HU}^{b}}{u}}$$

Since the variables a, b, u and v differ for each mechanical parameter, three different Eq. (2) for t/L are calculated for each printed resin. Averaging these three calculated t/L ratios for each material constant (E, σ_y_, σ_p_) gives the resulting t/L ratio used for the final bone model. In this way, all gathered mechanical parameters are included in the model.

### 3D-printed vertebral models

**Fig. 5:**
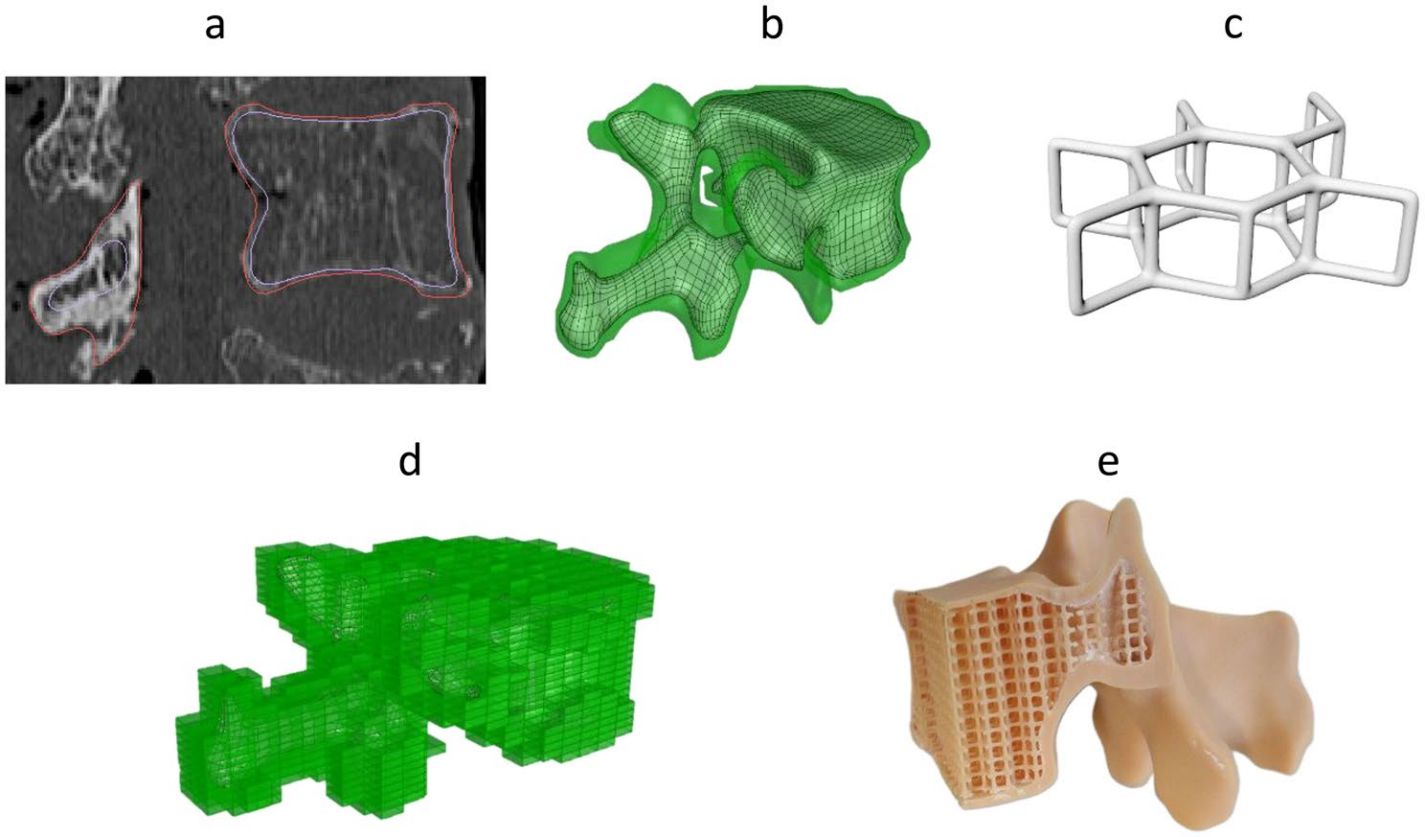
Workflow for creating the whole bone models of selected vertebrae. After segmentation (**a**) of the outer and inner cortical contours the two separate models are exported as STL files. The models were then imported and remeshed (**b**) in the CAD-Software Rhino (Rhino 7, Robert McNeel & Associates, Seattle, WA, USA). The unit cell (**c**) was then created using the calculated ratio of truss diameter and truss length (t/L) and the inner cortical contour is voxelized with the unit cells (**d**). Finally lattice and cortical shell were unified and exported as STL for 3D-Printing using stereolithography (**e**)

Three lumbar vertebrae from different donors were selected for replicating whole vertebral bones to cover different bone densities and different lumbar levels. Two of the selected donors had osteoporotic and one normal bone quality (Table 3). Both the outer and inner contours of the cortical bone were segmented using the threshold method. Care was taken to prevent overlapping contours (see Fig. 5a). Necessary corrections were made using the ‘Edit Contours’ software tool. The contours were exported from the segmentation software as STL-files and imported into the CAD-software for further processing. First, the models were remeshed with an element size of .8 mm (Fig. 5b). Model contours were checked again by re-importing the models into the segmentation software. This was followed by generating the parametrized unit cell (Fig. 5c) via HU of the vertebra (Fig. 5d). The lattice is then merged with the cortical bone using Boolean operations. Drain holes were integrated into the end plates and the vertebral processes to ensure proper cleaning of the non-polymerized resin.

The resulting 3D models (Fig. 5,e) were then prepared for printing using preprinting software (PreForm^®^, Formlabs, Sommerville, MA, USA) and sent to the printer. After printing, the models were thoroughly cleaned with isopropyl alcohol and post-cured under ultraviolet radiation and temperature according to the manufacturer’s instructions. As a pre-test for the pull-out tests, several vertebrae were made from the two previously analyzed resins and instrumented with pedicle screws. The vertebrae made with ClearV4 resin exhibited such brittle behavior that the models failed locally during instrumentation (pre-drilling, tapping, screw insertion). For this reason, whole vertebral models were printed out of Tough2000 V2 resin, which has a higher ultimate strain to avoid these issues (Table 1).

One lumbar vertebra with normal bone quality and two vertebrae with osteoporosis from different donors and different levels of the lumbar spine were selected for the final pull-out tests of the 3D-printed bones (Table 2). Each of these three human vertebrae was printed five times from Tough2000 V2 resin. The drain holes were sealed with adhesive tape to prevent penetration of the casting resin into the printed vertebrae during embedding.

**Table 2:** Specifications of the three lumbar vertebrae which were selected for 3D-printing. The selection is justified by ensuring the representation of different bone qualities and different vertebral levels

Donor	Level	Spongious HU	Spongious HU (screw side)	t/L
1	L3	118	93	0.17
3	L1	303	343	0.35
4	L4	62	58	0.13

### Statistical analyses

Descriptive statistics were examined using IBM SPSS Statistics 28 (IBM Corporation, Armonk, NY, USA). Statistical significance is defined with *P* < 0.05. Non-linear regression analysis using curve estimations based on power-law was used to determine relationships between the compressive properties (E, σ_y_, σ_p_) and the HU or the lattice parameter t/L. Linear as well as nonlinear (power, exponential) curve estimations were furthermore applied for analyzing the dependence of pull-out force and HU. Kruskal-Wallis test for independent samples were used to distinct between the of normal, osteopenic and osteoporotic vertebrae.

## Results

For better comprehensibility, first the results of all compression tests are described followed by the results from the pull-out tests on both the human vertebrae and the 3D-printed bones. The individual human vertebrae were classified as osteoporotic, osteopenic and normal based on DXA analysis (Table 3). Significant differences were found between normal and osteopenic (*P* < 0.001) as well as between normal and osteoporotic vertebrae (*P* < 0.001), but not between osteopenic and osteoporotic bones.

**Table 3:** Specifications of each body donor including age, sex, T-Score from Dual-Energy X-Ray imaging as well as the resulting osteoporosis classification as defined by the Word Health Organization. One vertebra of each marked (*) donor was selected for parametrization and 3D-printing

Donor (#)	Age (years)	Sex	T-score	Bone quality
1	97	female	-2.4	osteopenia
2*	79	female	-3.2	osteoporosis
3	81	male	0.8	normal
4	95	male	-3.2	osteoporosis
5*	78	male	1.1	normal
6*	91	male	-3.4	osteoporosis

### Compressive tests

The cancellous HU on the compressive specimen side ranges between 61 and 364 HU. An increase in the mechanical parameters was observed with increasing HU. The compressive modulus lies between 3.3 and 420.9 MPa, the compression limit between 2.0 and 4.3 MPa and the plateau stress between 0.1 and 4.2 MPa. According to Table 4, statistical correlations between the mechanical parameters and the HU were demonstrated via curve fittings based on power-law functions.

**Fig. 6:**
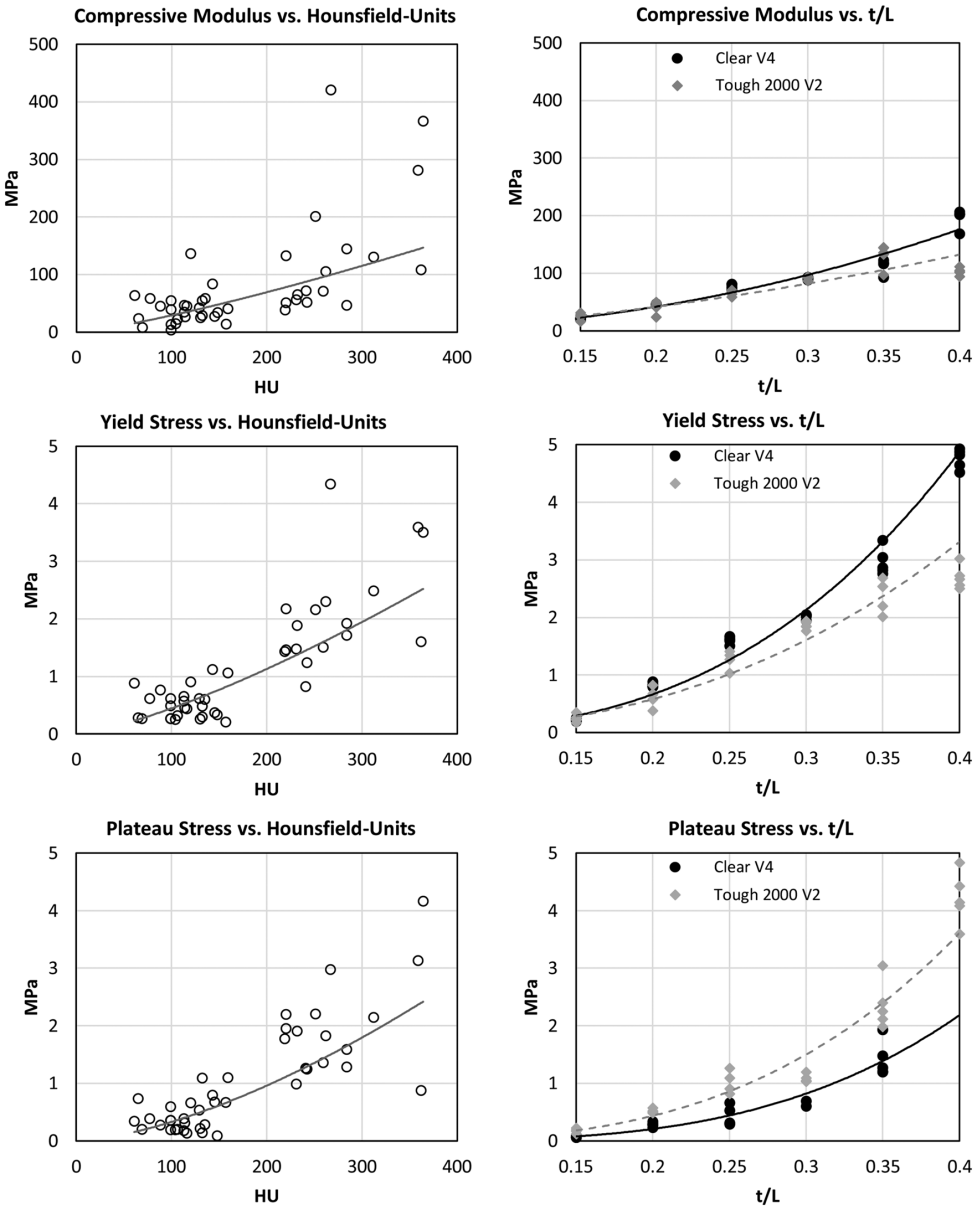
Scatterplots showing the compressive properties (Compressive Modulus, Yield stress and Plateau stress) of human bone specimens as functions of their Hounsfield units (left) and printed lattice specimens as functions of the ratio of truss diameter and truss length (right). All fittings are based on power laws. The lattice specimens were printed with two different resin materials (Clear V4 and Tough2000 V2, Formlabs, Sommerville, MA, USA

A correlation between the mechanical parameters of the printed lattice specimens was verified using the same method. For the Clear V4 material, E lies between 19.9 and 205.9 MPa, σ_y_ between 0.2 and 4.9 MPa and σ_p_ between 0.0 and 1.9 MPa. For the material Tough2000 V2, E lies between 16.2 and 144.3 MPa, the σ_y_ between 0.2 and 3.0 MPa and σ_p_ between 0.1 MPa 4.8 MPa. E of the samples made of Tough2000 V2 for t/L = 0.4 is approximately 100 MPa, which is around 10% below the average modulus of the samples with t/L = 0.35 (Fig. 6, top right). Furthermore, no σ_p_ could be determined for the samples made of ClearV4 for t/L = 0.4, as the samples bursted during the testing.

**Table 4:** Summary of all mechanical tests including human bone samples and 3D-printed lattice specimen made from photopolymer resins (Clear V4 and Tough2000 V2) using stereolithography. Non-linear regression analysis by means of power laws were calculated for each mechanical parameter and the Hounsfield units (bone samples) and fraction of strut diameter/strut length t/L (3D-printed specimens)

		Constant a	Exponent b	Regression Coefficient R²	P-Value
Human Bone vs. HU	Compressive Modulus	0.094	1.25	0.42	< 0.001
	Yield Stress	0.001	1.34	0.62	< 0.001
	Plateau Stress	0.000	1.54	0.58	< 0.001
Clear V4 vs. t/L	Compressive Modulus	1168.59	2.06	0.96	< 0.001
	Yield Stress	71.83	2.91	0.96	< 0.001
	Plateau Stress	67.13	3.65	0.91	< 0.001
Tough2000 vs. t/L	Compressive Modulus	609.53	1.66	0.87	< 0.001
	Yield Stress	33.32	2.51	0.93	< 0.001
	Plateau Stress	58.88	3.05	0.96	< 0.001

### Pull-out tests

**Fig. 7:**
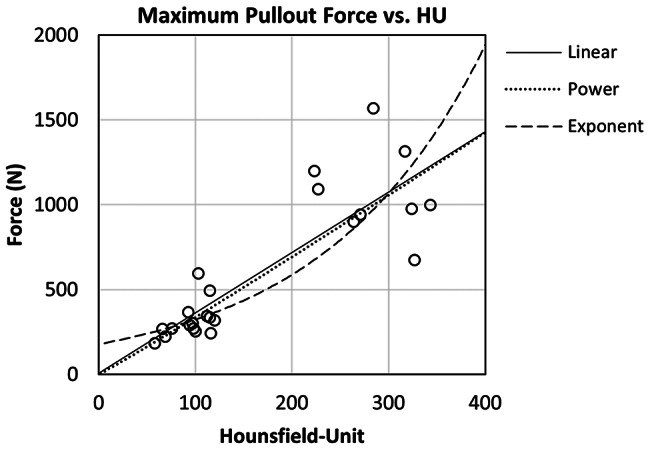
Scatter plot of the spongious Hounsfield unit of the instrumented vertebral hemispheres and their respective pullout forces

The HU of the instrumented vertebra hemispheres (*N* = 24) range between 58 and 343 HU. Corresponding F_max_ were between 183 and 1567 N (Fig. 7). Regression analysis containing linear as well as nonlinear curve fittings (Table 5) confirm statistically significant relationships between side-specific cancellous HU and F_max_.

**Table 5:** Summary of curve fits summarizing the relationship between maximum screw pull-out force and the Hounsfield unit of their corresponding vertebral half

Curve Fit	a	b	*R*²	*P*-value
Linear (y = a*x + b)	5.65	3.56	0.73	< 0.000
Power (y = a*x^b^)	2.77	1.04	0.83	< 0.000
Exponential (y = a*e^b^)	177.75	0.01	0.78	< 0.000

**Fig. 8:**
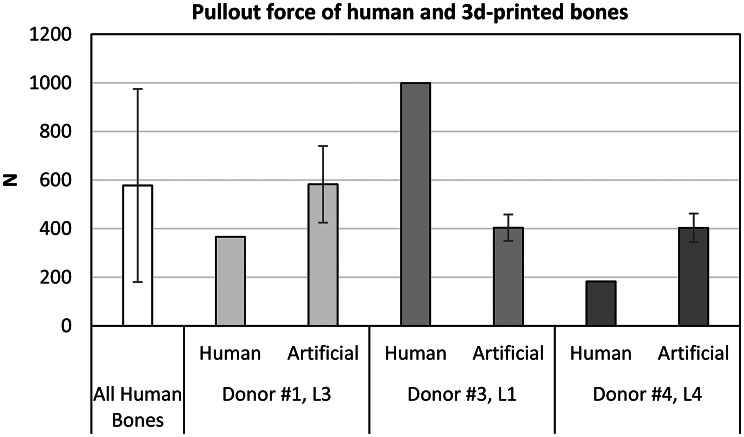
Pullout forces of 3 selected human vertebrae and the mean value (error bars show standard deviation) of each group of their replicas (*N* = 5)

The averaged F_max_ of the 3D-printed replicas (donor #1, L3 and donor #4, L4) are 200 N higher than their respective human original. The mean F_max_ of the replicas of donor #3 L1 is 600 N lower compared to their human originals (Fig. 8).

## Discussion

This study aimed at developing a parametrizable structure as a substitute for spongious bone in vertebral bones for 3D-printing individualized anatomical models. This was done by designing and manufacturing hexagonal lattice specimen and comparing them with human trabecular bone by means of compressive mechanical testing. The approach was then finalized by recunstructing whole vertebral bones, implementing the parametric lattice, and biomechanically evaluating said 3D-printed models with pedicle screw pull-out tests.

### Compressive tests

Vertebral cancellous bone HU ranges from 61 to 364 HU. The distribution of HU indicates a distinction between the two main groups. Those above 200 HU represent vertebrae with normal bone density and those below 200 HU are assumed to be either osteopenic or osteoporotic. This is consistent with previous studies investigating the relationship between bone quality and HU from diagnostic CT [32–37].

The DXA analysis was only able to assess bone density in the lumbar vertebral bodies. Based on the DXA results, bone quality evaluation across all six donors revealed that two had normal bone quality, one was classified as osteopenic, and three were osteoporotic.

Our mechanical parameters show strong concordance with comparable data reported in the literature [38–41]. Öhman-Mägi et al. [42] reviewed data on the mechanical properties of cancellous bone from vertebral bodies. They found elastic moduli ranging from 1 to 976 MPa. They also found yield stresses ranging from 0.1 to 14 MPa. Our values are relatively low, likely due to the geriatric age of our body donors and the consequently reduced bone quality.

σ_y_ and σ_p_ (R² = 0.62 and R² = 0.58 respectively) showed higher R² values and therefore better model quality than E (R² = 0.42) (Table 4). However, the variance in our data could be due to the fact that we did not measure the exact HU of the samples, but the averaged HU of the vertebral halves from which the samples were taken. Additionally, biological noise as well as methological errors could alse contribute to variances within the presented statistical models. The exponent of the power equation lies within 1.3 and 1.5 which corresponds well with experimental findings comparing HU and compressive mechanical properties of human bone from various anatomical sites [43]. So, plausible and practically useful correlations for lattice dimensioning can be derived by using power functions for predicting the mechanical properties of spongious bone. Strong correlations between the lattice parameter t/L and the mechanical properties of the lattice samples were demonstrated based on power-law functions (Table 4). Both material properties matched the properties of the human samples very well. Fig. 6 shows that the mechanical parameters scatter more with increasing t/L. Initial tests showed that cylindrical specimens could be printed best when aligned horizontally on the building platform of the printer, meaning that the longitudinal axis of the cylindrical samples is orthogonal to the printing direction.

At higher t/L, component distortion during post-processing could not be completely ruled out, so that the plates on the front sides of the samples (Fig. 4, right) are no longer parallel to each other. This was especially noticeable in the samples made of the material Tough2000 V2. Samples made of Clear V4 resin further showed incremental failure of the lattice layers at low values of t/L. There was no constant plastic failure zone at high strains as was the case with the bone samples. Especially the samples from Though2000 V2 material (Fig. 2) showed viscoelastic material behavior in the linear range as observed for bone specimens (Fig. 4). But it has to be noted, that bone subtstance itself has much higher material properties with an elastic modulus of around 17 GPa in femoral bone [44] than the printed resins with around 2–3 GPa (Table 1). Those differences must be compensated by increasing the relative lattice density.

Lattice structures are primarily used in lightweight engineering to maximize mechanical properties while minimizing component mass. According to Ashby, lattice structures are classified as tensile or bending-dominated structures depending mainly on the type of unit cell (e.g. octet-truss unit-cell, hexagonal unit cell). The exponent in the relationship between relative density and stiffness allows a distinction between bending and tension-dominated structures [45]. Vertebral cancellous bone is a largely open-cell structure with a relatively low bone volume content of 8.15% [28] and is therefore a bending-dominated structure [27]. Our t/L ratio is proportional to its relative density (Fig. 3, right) and closely matches the relative density of vertebral cancellous bone, ranging from 4 to 12% [28]. We can thus show that the chosen hexagonal lattice structure with its bending-dominated properties is well suited for mimicking vertebral cancellous bones, especially for bones with poor bone quality. Future works should focus not only on other cellular structures and lattice types, but also on the local manipulation of individual cells and cell structures in order to replicate the heterogeneity of the cancellous bone as accurately as possible, thereby transferring the presented model approach to other anatomical regions and better bone quality.

### Pull-out tests

Similar to the compression specimens with previous investigations, we found rather low F_max_ in the human specimens [46–48] Which is related to their poor bone quality [49, 50]. The regression analysis of F_max_ vs. HU shows significant models for each curve fit, with the power-law function showing highest R² = 0.83. Previous studies could also show significant correlations between HU and screw anchorage. Matsukawa et al. [51] examined the Regional HU within the screw trajectory in vivo. They found significantly lower summarized HU for the loosened screws compared to the fixed ones. Similar findings were published by Zou et al. [52]. Another group around Wichmann [53] measured the mineral content by means of quantitative CT within different regions alongside the screw trajectory and compared it to the maximum pullout force in vivo. Best correlations were found by comparing the pullout force with the mineral content inside the pedicle. Their pullout forces range between roughly 100 and 1300 N which is in good agreement with our data presented in Fig. 7.

The above-mentioned grouping of the data points regarding bone quality is also recognizable here. Our data show that a reduced bone quality can be assumed for F_max_ of less than 500 to 600 N.

While instrumenting the printed vertebral replicas, it was determined in preliminary tests that post-processing (cleaning with isopropanol and post-curing according to the manufacturer’s specifications under temperature and ultraviolet radiation) of the vertebrae significantly influenced the print quality. This may explain the variance in F_max_. We found on average 200 N higher F_max_ on the replicas of vertebrae #1, L3 and #4, L4 than compared to their human counterparts, which both had low bone quality. In our opinion, the most plausible explanation for this is that the cortical bone in the pedicle is too thick, which could be caused by errors during segmentation or during post-processing. Since about 60% of the pull-out force depends on the cortical fixation of the screw within the pedicle [54, 55], the unintended decrease of the pedicle diameter within the vertebral replicas increased their F_max_.

Conversely, the high pull-out force of specimen #3, L1 failed to be replicated by the printed resin, even though it has an appropriately dimensioned lattice. These observations confirm the argument of cortical anchorage. These lower pull-out forces in comparison to the human model can therefore be explained by the difference in strength between synthetic resin (modulus of elasticity 1.9 GPa, Table 1) and cortical bone (modulus of elasticity around 18 GPa [56]).

Thus, we conclude that the chosen model approach is well suited for replicating bones with low bone quality. For the replication of healthy bones or bones with pathologically increased mechanical properties (e.g. due to osteoarthritis, necrosis, tumors), other model materials, material combinations (e.g. short fiber reinforcements), printing technologies (e.g. Material-Jetting) or lattice structures should be investigated.

### Limitations

The presented bone models were based on HU from opportunistic CT-scans. For this reason, a major limitation of our study is the transferability of our specific parameterization results to other clinical CT-scans, since HU from CT depends on the machine type, tube voltage or current used. A quantification of the HU could be performed retrospectively for bone replications based on other scanners or parameters, e.g. by external calibration of the scans. Although a direct transfer of the presented dependence of HU and mechanical parameters is not possible due to said reasons, the parametrization can also be done by referencing the power laws of the 3D-printed lattice to any mathematically described curve fit between trabecular bone and the volumetric bone mineral content (e.g. [57]). This will work as long as one can provide the volumetric bone mineral density of the vertabral bones to be recreated by our model approach. Hence, the presented workflow is transferable to clinical application by parametrizing the presented power-law of the printed lattice with literature data regarding the relationship between volumetric bone mineral density and mechanical parameters since evaluating the lattice structure is unrelated to the structural-functional relationship of the bone to be replicated.

High t/L specimens in particular showed warping during printing, which may have an influence on the internal stress conditions within the specimens affecting the mechanical properties. All overhanging structures require reinforcement with support structures during SLA printing. These supports must be removed after printing, which, depending on the orientation within the build volume, can potentially damage the cylindrical specimens. Positioning the specimens horizontally offered the best compromise between reliable printing and minimal post-processing. Besides part geometry, warping is also highly influenced by the material used.

Each human specimen was printed five times and underwent a pull-out test, so we tested the in vitro structure five times per vertebral body with the same (replicated) bone geometries by using the pull-out test. We found large variations in F_max_, which can be explained by irregularities in printing, which are estimated to be rather small, as the lattice in the vertebrae is supported from all sides in contrast to the compression specimens. Furthermore the mean standard deviation off all Though2000 V2 compressive specimen is about 13%. As only 40% of the pull-out force is related to the spongious bone within the vertebral body [54], only a fraction of the above mentioned deviation will influence the resulting F_max_. Hence, we believe that the greatest influence on the variation of F_max_ is the chosen freehand implantation technique. In the future, deviations in the screw trajectories could be avoided by using individualized drilling templates.

Even though the inner and outer cortical contours of both the mask and the resulting 3D-object were thoroughly checked during segmentation, we did not measure the cortical thickness of neither bone nor reconstructed models. Errors during segmenting the cortex could lead to significant changes of the structural stiffnes. This is why future studies should address the cortical thickness by validating the reconstruction algorithm by means of using micro-CT, which could then be used for validating the cortical thickness from reconstructions based on clinical imaging resolution.

## Conclusion

This study presents a method for replicating individual human vertebrae using 3D-printing. A hexagonal lattice structure was designed with mechanical properties that can be precisely tuned by adjusting the ratio of rod thickness to rod length. Uniaxial testing established mathematical relationships between the mechanical properties, the HU from diagnostic CT data, and the lattice parameter t/L. By integrating these lattice structures within CT-reconstructed bone geometry, entire vertebrae can be accurately reproduced using SLA printing. This individualized approach was validated through uniaxial pull-out tests of pedicle screws on both human specimens and their printed replicas, demonstrating its utility in simulating implant anchorage in vertebrae with low bone quality. Unlike generic bone models, our method enables the creation of anatomically specific, reproducible replicas directly from patient CT data, allowing for precise in vitro testing and the development of customized treatment strategies tailored to each unique anatomy. 

## Data Availability

The datasets used and/or analysed during the current study are available from the corresponding author on reasonable request.

## Abbreviations

3D: Three dimensional
t: Rod thickness of the lattice
L: Rod length of the lattice
µCT: Mocir computed tomography
HU: Hounsfield-Unit
CT: Computed tomography
T7: Seventh thoracal vertebrea
L5: Fifth lumbal vertebrea
DXA: Dual-Energy-X-Ray
Fig: Figure
F_max_: Maximum pullout force
MPa: Megapascal
E: Compressive modulus
σ_y_: Compressive stress
σ_p_: Plateau stress
CAD: Computer aided design
SLA: Stereolithography
y: Algebraic Variable
x: Algebraic Variable
a: Product within power equation
b: Exponent within power equation
Tab: Table
p: Statisical p-value
vs.: Versus
N: Number
R^2^: Regression coefficient
e.g.: For example
